# Evaluation of long-term immunity following inoculation with highly diverse orthomarburgvirus isolates in Egyptian rousette bats (*Rousettus aegyptiacus*)

**DOI:** 10.1128/jvi.00848-25

**Published:** 2025-08-11

**Authors:** Jessica A. Elbert, Amy J. Schuh, Brian R. Amman, Jonathan C. Guito, James C. Graziano, Tara K. Sealy, Elizabeth W. Howerth, Jonathan S. Towner

**Affiliations:** 1 Department of Pathology, College of Veterinary Medicine, University of Georgia 1355, Athens, Georgia, USA; 2 Viral Special Pathogens Branch, Division of High-Consequence Pathogens and Pathology, National Center for Emerging and Zoonotic Infectious Diseases, U.S. Centers for Disease Control and Prevention 1242, Atlanta, Georgia, USA; 3 U.S. Public Health Service Commissioned Corps 33426 , Rockville, Maryland, USA; The Ohio State University, Columbus, Ohio, USA

**Keywords:** Egyptian rousette bat, Marburg virus, Ravn virus, viral coinfection, immunity, filovirus, reservoir

## Abstract

Viral coinfections and their impact on long-term immunity represent an understudied area in disease ecology and infectious disease research. Coinfections can influence the host’s susceptibility to future infections, alter host and pathogen population dynamics, modify infection and shedding patterns, impose evolutionary pressures, and affect the risk of zoonotic spillover. Egyptian rousette bats (ERB; *Rousettus aegyptiacus*; common name: Egyptian rousettes) are a natural reservoir host for Marburg virus (MARV) and Ravn virus (RAVV), as well as a vertebrate reservoir for Kasokero virus (KASV) and a putative reservoir for Sosuga virus (SOSV). Viral coinfections have been documented in numerous free-ranging bat species as well as ERBs, raising questions about how these interactions influence immune responses, viral shedding, and pathogen maintenance within the natural host population. This is particularly critical given the genetic diversity among co-circulating viral species (e.g., MARV and RAVV) and the potential implications for the development of protective immunity and subsequent viral inoculation outcomes. In this study, ERBs previously infected with MARV alone or KASV + MARV were inoculated approximately 8 months later with homotypic (MARV) or heterotypic (RAVV) orthomarburgvirus isolates. The results demonstrated no viral replication or shedding post-inoculation, and all bats displayed strong secondary immune responses consistent with sterilizing immunity. These findings suggest that both MARV monoinfection and KASV + MARV coinfection confer robust protection against reinfection, regardless of the viral isolate. This research enhances our understanding of immune responses during viral coinfections in bats and their potential role in mitigating zoonotic pathogen spillover.

**IMPORTANCE:**

Long-term immunity elicited in bats during coinfection (i.e., simultaneous infection) with viruses they naturally host is not well understood. These interactions could affect susceptibility to subsequent reinfection and pathogen spread. Egyptian rousette bats, natural hosts for several pathogenic zoonotic viruses, including Marburg and Kasokero viruses, can be found multiply infected in the wild, but the immune consequences of being coinfected remain unclear. Here, bats previously infected with either Marburg virus alone or with both Kasokero and Marburg viruses were later challenged with Marburg virus or the related Ravn virus. No reinoculated bats showed signs of virus replication, and all mounted strong immune responses. These results suggest that this coinfection combination still provides robust protection against reinfection, even with diverse orthomarburgviruses. This study helps improve our understanding of how bats manage viral coinfections and may inform how these interactions influence zoonotic spillover risk.

## INTRODUCTION

Understanding the effects of viral coinfections on long-term immunity is a minimally explored area in disease ecology and infectious disease research, and it is a topic of heightened importance in reservoir hosts that harbor zoonotic pathogens with significant public health impacts. Coinfections—instances where a host is either infected simultaneously or in close succession with two or more pathogens—can alter the host’s susceptibility to future infections, affect both host and pathogen populations, create evolutionary pressures, and influence the risk of zoonotic spillover (1–5). Gaining more insight into the consequences of pathogen coinfections is crucial for understanding the drivers of zoonotic pathogen transmission and for developing strategies to prevent future outbreaks (6, 7).

With over 1,400 recognized species, bats (order Chiroptera) constitute approximately 20% of all mammalian species, second only to rodents in terms of diversity (8). They are known to host an incredible diversity of viruses, with sequences of over 22,000 isolates detected from more than 200 viruses across 32 viral families isolated as of May 2024 (9). A significant portion of bat viral diversity remains uncharacterized, with discovery efforts often prioritizing viral families with known zoonotic potential, such as *Coronaviridae*, *Filoviridae*, and *Paramyxoviridae* (5, 9–11). The Egyptian rousette bat (ERB; *Rousettus aegyptiacus*; common name: Egyptian rousettes) is a natural reservoir host for Marburg and Ravn viruses (MARV; RAVV; family *Filoviridae*; genus *Orthomarburgvirus*) (12–14), as well as a vertebrate reservoir for Kasokero virus (KASV; family *Orthonairoviridae*, genus *Orthonairovirus*) (15) and a presumed reservoir for Sosuga virus (SOSV; family *Paramyxoviridae*, genus *Pararubulavirus*) (16–19).

MARV and RAVV are the only two known virus members of the species *Orthomarburgvirus marburgense* and are causative agents of Marburg virus disease (MVD), a severe disease that typically emerges in sub-Saharan Africa and is characterized by human-to-human transmission and high case fatalities (20). MARV was first identified in 1967 following laboratory outbreaks in Marburg and Frankfurt, Germany and Belgrade (former Yugoslavia) linked to exposure to African green monkeys imported from Uganda (21). To date, 18 known orthomarburgvirus outbreaks have occurred, most recently in Tanzania in January 2025 (22). Many MARV outbreaks have been epidemiologically linked to exposure to ERBs through gold mining activities or other human encroachment into ERB habitats, including tourism (23–30). RAVV was first identified in 1987 following a fatal case in a tourist visiting Kitum Cave, Mount Elgon National Park, Kenya (28). Designated as a distinct virus within the species in 1996, RAVV has since been identified in two subsequent outbreaks, the latest in Uganda in 2007 (12, 27, 28).

Experimental studies have provided insight into the natural history and pathogenesis of orthomarburgvirus infection in its reservoir host (14, 31–36). MARV-inoculated ERBs have limited and subclinical disease characterized by viremia, broad viral tissue dissemination, viral shedding in saliva, feces, and urine, and mild hepatitis characterized by multifocal aggregates of mononuclear cells and mild hepatocellular necrosis with an increase in alanine aminotransferase (ALT) (14, 31, 32, 34, 36, 37). MARV IgG antibodies peak by 28 days post-infection (DPI) and usually fall below the threshold of seropositivity by 3 months post-infection (MPI) (31, 32, 35, 36). Horizontal transmission of infectious disease between experimentally inoculated and naïve, co-housed ERBs has been documented in a laboratory setting (35), as well as robust short- (31) and long-term (33) immunities to reinfection upon experimental homotypic MARV inoculation 48 DPI and 2 years after initial infection, respectively. Vertical transmission has not been observed in MARV PCR-positive dams carrying newborn pups (12).

Ecological and experimental studies have shown the ERB to be a natural vertebrate reservoir for KASV, a human-pathogenic virus maintained in an enzootic transmission cycle with ERBs and *Ornithodoros* (*Reticulinasus*) *faini* ticks (15, 38–41). Experimental KASV-infected bats develop mild to moderate, acute viral hepatitis, which first presents at 3 DPI and is cleared by 20 DPI, with viral shedding primarily detected in blood and in oral and rectal swabs and urine (39, 40, 42). Viral replication occurs in the liver, spleen, lymph nodes, and tongue, with KASV RNA cleared from the spleen and liver by 6 DPI (42).

The prevalence of coinfections in free-ranging bat populations is poorly understood, typically noted incidentally rather than through systematic study (5). A recent retrospective analysis of ERB tissues from bats living in Kitaka Mine, Uganda and in Sierra Leone showed that 2.47% of juveniles infected with MARV were also infected with SOSV, with one bat coinfected with MARV, SOSV, and Yogue viruses (family Orthonairoviridae, genus *Orthonairovirus*) (43). Worldwide surveillance efforts have documented widespread viral coinfections in bats, with up to 42% of sampled bats in China coinfected with at least two viruses spanning 12 viral families (44) and similar findings reported globally involving diverse viral combinations, including coronaviruses, astroviruses, and paramyxoviruses, among others (45–66). These studies underscore the remarkable virological diversity in bats and their role as reservoirs for multiple, often concurrent, viral infections, highlighting the need for comprehensive surveillance to better understand the prevalence, complexity, and ecological significance of coinfections both within individual animals and larger ecosystems.

A recent experimental study found that viral coinfection in ERBs can modulate viral shedding dynamics and subsequent antibody development, suggesting alterations to long-term immunity (19). ERBs experimentally infected with SOSV + MARV had significant reduction in the duration of MARV shedding, whereas ERBs coinfected with KASV + MARV had significantly increased peak magnitude and duration of MARV viremia and oral shedding, resulting in significantly higher cumulative MARV shedding in KASV + MARV coinfected bats (19). Prior to this study, experimental studies on coinfections in bats were limited to investigations into interactions between coronaviruses and non-viral pathogens. A 60-fold increase in coronavirus RNA was observed in the intestines of *Myotis lucifugus* bats coinfected with *Pseudogymnoascus destructans*, the fungal pathogen that causes white nose syndrome (67). Given the frequent occurrence of viral coinfections in free-ranging populations and the high contact rates within densely populated roosts that facilitate repeated viral exposure, the impact of coinfection on responses to subsequent viral exposures remains poorly understood and warrants further investigation.

Despite being within the same viral species, the full genomic sequence of RAVV differs by up to 21% from MARV and the amino acid sequence of the RAVV glycoprotein (GP) differs by ~22% from MARV GP (12, 68) and, as such, may generate antigenically distinct pathogen-associated molecular patterns (PAMPs). Therefore, the more sensitive and specific actions of effector T cells and class-switched, affinity-matured antibodies that would provide protective immunity for homotypic inoculation may not be able to block infection of a heterotypic isolate, and viral replication and shedding may be seen. The extent to which heterotypic inoculation will allow viral replication and shedding in a previously monoinfected or coinfected ERB is currently unknown.

In this study, we assess whether inoculation with a homotypic or heterotypic orthomarburgvirus isolate (MARV or RAVV, respectively) influences viral reinfection, replication, and shedding in either previously MARV-monoinfected or KASV + MARV-coinfected ERBs. Additionally, we assess whether coinfection with KASV + MARV confers long-term protective immunity against reinfection, replication, and shedding by inoculating ERBs that had been experimentally infected ~8 months prior during a previous coinfection study (19). Data for RAVV-monoinfected bats were taken from a recent characterization of RAVV viral shedding dynamics in experimentally infected ERBs (69) and included as a control for baseline Ravn virus viral shedding dynamics. Following inoculation, MARV and RAVV detection in the blood and viral shedding from the oral and rectal mucosa is monitored for 10 days, and MARV IgG antibody responses are monitored for 21 days. Here, we show that no bats previously infected in any group have evidence of MARV or RAVV replication or shedding, regardless of prior infection history. Furthermore, all bats developed virus-specific secondary immune responses, demonstrating that infection with MARV induces sterilizing immunity (defined herein as absence of detectable viremia and viral shedding for 10 consecutive days post-inoculation) against orthomarburgvirus reinfection with a genetically distinct virus, even when the initial MARV infection was initiated in the context of KASV coinfection.

## MATERIALS AND METHODS

### Virus

Four log_10_ 50% tissue culture infective doses (TCID_50_) of a MARV isolate (200704852 Uganda bat, termed MARV371; second passage on Vero E6 cells) obtained from a naturally infected ERB (371bat) and a RAVV isolate (200704669 Uganda bat; second passage on Vero E6 cells) obtained from a naturally infected ERB (188bat), both collected during a 2007 orthomarburgvirus outbreak ecological investigation at Kitaka Mine in southwestern Uganda, were prepared in 0.25 mL of sterile Dulbecco’s modified Eagle’s medium (DMEM) (12).

### Bats

Procedures conducted with infectious orthomarburgvirus or with infected bats were performed at the CDC under biosafety level 4 (BSL-4) laboratory conditions in accordance with Select Agent regulations (Animal and Plant Health Inspection Service and Centers for Disease Control and Prevention 2014). All investigators and animal handlers followed strict BSL-4 safety and infection control practices (70). Thirty-six adult ERBs (12–14 months of age) were used in this study. All ERBs were captive-born bats and managed as previously described (36, 69).

### Experimental design/bat groups

Twenty-four ERBs were previously utilized in an experimental infection study at CDC, investigating viral shedding dynamics upon coinfection of ERB bats with KASV + MARV (19). In that study, ERBs were infected with 4 log TCID_50_ of KASV (−6 DPI) and/or MARV (0 DPI), underwent non-destructive sampling, and were maintained for 8 months in BSL-4 laboratory conditions prior to the onset of the experimental study described herein. Twelve ERBs were undergoing a concurrent experimental study characterizing viral shedding dynamics of Ravn virus and will be highlighted here as Group 5, a control group for baseline Ravn virus viral shedding dynamics in primed ERBs (69). Experimental groups are outlined in Table 1.

**TABLE 1:** Experimental group designations, inoculation history, and experimental aims

Group	No. of ERB (*n* = 36)	Sex^*a*^	Prime inoculation: March 2023	Challenge inoculation: October 2023	Prime inoculation: October 2023	Experimental aim
1	6	4 M, 2 F	MARV	MARV	X^*b*^	Characterization of long-term immunity upon homotypic inoculation
2	6	4 M, 2 F	KASV + MARV	MARV	X	Assess role of viral coinfection on long-term immunity upon homotypic inoculation
3	6	3 M, 3 F	MARV	RAVV	X	Characterization of long-term immunity upon heterotypic orthomarburgvirus inoculation
4	6	3 M, 3 F	KASV + MARV	RAVV	X	Assess role of viral coinfection on long-term immunity upon heterotypic orthomarburgvirus inoculation
5	12	6 M, 6 F	X	X	RAVV	Establish baseline RAVV viral shedding dynamics

^*a*^ M, male; F, female.

^*b*^ X, procedure not performed.

The experimental design is outlined in Table 2. ERBs from a previous coinfection study (19) (groups 1–4) and naïve ERBs (Group 5) (69) were acclimated in the BSL-4 laboratory for 7 days before the beginning of the study (acclimation study phase). Baseline blood samples, body weights, and temperatures were taken prior to inoculation. At 0 days post-inoculation (DPI), bats in groups 1 and 2 were inoculated subcutaneously under isoflurane anesthesia with the above-described MARV inoculum in the caudal abdominal region. Bats in groups 3–5 were inoculated subcutaneously under isoflurane anesthesia with the above-described RAVV inoculum in the caudal abdominal region. As data from historical MARV control ERBs from previous studies were available, control ERBs were not utilized in this study.

**TABLE 2:** Overview of experimental design^*a*^

DPI	Group 1	Group 2	Group 3	Group 4	DPI	Group 5
−236 (KASV infection: 3/2/2023, MARV infection: 3/8/2023) (19)	Prime MARV inoculation	Prime KASV + MARV inoculation	Prime MARV inoculation	Prime KASV + MARV inoculation	−236 (69)	X
-7	BSL-4 acclimation				−7	BSL-4 acclimation
-1	Pre-bleeds Body weights				−1	Pre-bleeds Body weights
0 (10/24/2023)	MARV inoculation	MARV inoculation	RAVV inoculation	RAVV inoculation	0 (10/24/2023)	Prime RAVV inoculation
1–10	Weekly body weights Daily temperatures Daily collection of blood, oral swabs, and rectal swabs				1–21	Weekly body weights Daily temperatures Daily collection of blood, oral swabs, and rectal swabs
11–21	Weekly body weights Weekly blood collection					
22	Euthanasia				22	Euthanasia

^*a*^ DPI, days post-inoculation; X, procedure not performed.

### Specimen collection

Specimen collection has previously been described in detail (34–36). Blood (whole, nonheparinized; 10 and 21 µL for RT-qPCR and serology, respectively) was taken on −1 DPI daily from 1 to 10 DPI and then on 14 and 21 DPI from the cephalic wing vein using a sterile lancet (C&A Scientific, Manassas, VA, USA). Blood was tested for the presence of orthomarburgvirus RNA by RT-qPCR through 10 DPI, and orthomarburgvirus IgG antibody responses were monitored weekly through 21 DPI. The oral mucosa was sampled daily through 10 DPI by swabbing simultaneously with two polyester-tipped applicators inside of the bat’s mouth (Fisher Scientific, Grand Island, NY, USA). After sampling, one oral swab was immediately placed in either a deep-well plate with 500 µL of MagMAX lysis buffer solution (Life Technologies, Grand Island, New York, USA) for nucleic acid extraction and RT-qPCR analysis, and one oral swab was placed in sterile viral transport medium for attempted virus isolation of any orthomarburgvirus RNA positive swabs. A temperature probe covered with a plastic sheath (MABIS Healthcare, Waukegan, Illinois, USA) was used to measure the rectal temperature of each bat through 10 DPI. The plastic sheath was then cut and placed into a deep-well plate with 500 µL of MagMAX lysis buffer solution (Life Technologies) for nucleic acid extraction and RT-qPCR analysis.

### Euthanasia

At 22 DPI, all bats were euthanized under anesthesia via an overdose of isoflurane, followed by cardiac exsanguination. Cardiac blood was collected and retained.

### Nucleic acid extraction

Nucleic acid was extracted from blood, oral swab, and rectal probe covers using the MagMAX Pathogen RNA/DNA Kit (Thermo Fisher Scientific, Waltham, MA, USA) on the KingFisher Apex 96 Deep-well Head Magnetic Particle Processor (Thermo Fisher Scientific).

### RT-qPCR

RT-qPCR procedures have previously been described in detail (35, 36). Reverse-transcribed orthomarburgviruses and ERB beta-2-microglobulin (B2M) RNA were detected on the CFX Opus 96 Real-Time PCR System (Bio-Rad, Hercules, CA, USA) using the Luna Probe One-Step RT-qPCR 4× Mix with UDG (New England Biolabs Inc, Ipswich, MA, USA), with amplification primer and reporter probes targeting the orthomarburgvirus viral protein 40 (VP40) gene (forward primer: GGACCACTGCTGGCCATATC, reverse primer: GAGAACATITCGGCAGGAAG, probe 1: 56-*FAM*-ATC CTA AAC-*ZEN*-AGG CTT GTC TTC TCT GGG ACT T-*3IABkFQ*, probe 2: 56-*FAM*-ATC CTG AAT-*ZEN*-AAG CTC GTC TTC TCT GGG ACT T-*3IABkFQ*) and the ERB B2M gene (forward primer: CAGCAAGGACTGGTCTTTCTAT, reverse primer: CCTCCATGATGCTGGTTAGTT, probe: *FAM*-TTC ACA CGG-*ZEN*-CAG CTG TAC TCA TCC-*IABkFQ*), respectively. This assay was designed to detect a conserved sequence of VP40 present in all known species of orthomarburgvirus, including Ravn virus (68). Relative MARV and RAVV TCID_50_eq/mL (blood, oral, and fecal specimens) were interpolated from standard curves generated from serial dilutions of the titrated 371bat MARV isolate and 188bat RAVV isolate spiked into appropriate biological specimens.

### Serology

As previously described (33, 35, 36), enzyme-linked immunosorbent assay plates were coated with 50  ng per well of purified recombinant Marburg Angola nucleoprotein (NP) or Reston NP expressed in *Escherichia coli* (GenScript, Piscataway, NJ, USA) diluted in PBS containing 1% thimerosal. The plates were incubated overnight at 4°C and then washed with PBS containing 0.1% Tween-20 (PBS-T). A 1:100 dilution of gamma-irradiated bat whole blood in master plate diluent (PBS containing 5% skim milk powder, 0.5% Tween-20, and 1% thimerosal) was then added to the first well, and fourfold serial dilutions in serum diluent (PBS containing 5% skim milk and 0.1% Tween-20) were performed through 1:6,400. After incubating for 1 h at 37°C, the plates were washed with PBS-T, and bound antibody was detected using a 1:11,000 dilution of anti-goat bat IgG (Bethyl Laboratories, Montgomery, TX, USA) in serum diluent. Following incubation with the secondary antibody for 1 h at 37°C, the plates were washed twice with PBS-T, and the 2-Component ABTS Peroxidase System (KPL, Gaithersburg, MD, USA) was added. The substrate was allowed to incubate for 30 min at 37°C before reading the plates on a microplate spectrophotometer at 410 nm. The adjusted sum OD values were calculated by subtracting the ODs at each fourfold dilution wells coated with Reston NP from their corresponding wells coated with Marburg Angola NP and then linearly transforming the values using the min-max normalization method. The threshold for seropositivity was set at 0.07 after in-house assay optimization. The cut-off value for assay seropositivity was determined by calculating the mean adjusted sum OD value plus 3–5 standard deviations (SDs) of 38 MARV-naïve ERBs.

## RESULTS

### No evidence of orthomarburgvirus replication or shedding

Consistent with previous studies that describe short- and long-term protective immunities against MARV infection, replication, and shedding in previously monoinfected ERBs (31–33), none of the either MARV-monoinfected or MARV + KASV bats (groups 1–4) developed detectable viremias or shed viral RNA when inoculated with either MARV or RAVV throughout the 10-day specimen collection period. Sample collection was originally scheduled to extend through the end of the study; however, since all samples were universally negative for 10 consecutive days (1–10 DPI), daily sampling was ceased to avoid unnecessary stress on the animals. Due to the uniform negativity of the samples, tissues were not collected at necropsy for evaluation of MARV RNA viral loads. Data for RAVV monoinfected bats (Group 5) were taken from a recent characterization of RAVV viral shedding dynamics in experimentally infected ERBs (69) and included as a control for baseline Ravn virus viral shedding dynamics in primed ERBs. As described previously (69), viremia was present in all Group five bats, peaking on 5 DPI and cleared by 13 DPI and positive oral and rectal swab samples through 21 DPI.

### Rapid immune response upon inoculation

**Fig 1:**
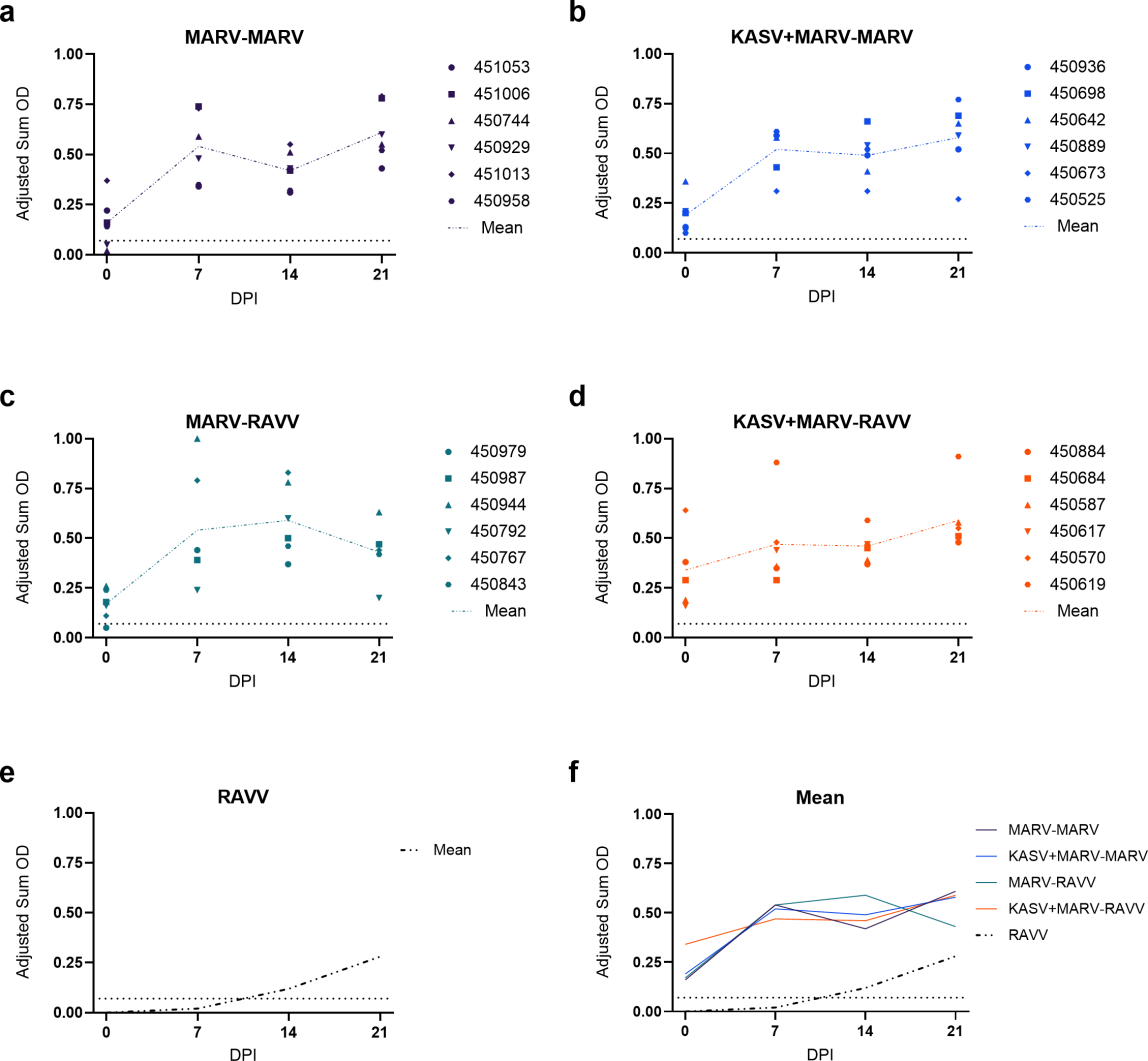
MARV IgG antibody responses of Egyptian rousette bats according to group (a–d). Orthomarburgvirus IgG antibody responses of experimentally inoculated bats as detected by enzyme-linked immunosorbent assay with purified recombinant nucleoprotein of the Angola isolate of MARV expressed in *Escherichia coli*. Data for RAVV monoinfected bats (e) were taken from a recent characterization of RAVV viral shedding dynamics in experimentally infected ERBs (69) and included as a control for baseline Ravn virus shedding dynamics in primed ERBs. Means across all groups are compared in (f). The black dotted line represents the cutoff value of the assay (MARV seropositive ≥ 0.5). DPI = days post-inoculation.

During the previous coinfection study, all bats (12/12 KASV + MARV and 12/12 MARV monoinfected ERBs) seroconverted to MARV by the study end (21 DPI) (19). By the initiation of this study at ~8 MPI, 21/24 (87.5%) of the previously infected ERBs maintained MARV IgG antibody levels above the threshold of seropositivity. Upon viral inoculation, all groups developed a robust MARV IgG antibody response by day 7, which was maintained through study end (21 DPI) (Fig. 1). There were no statistically significant differences in MARV IgG antibody levels between the four inoculation groups (*F*_3,12_ = 0.02291; *P* = 0.9950).

### No evidence of clinical disease

**Fig 2:**
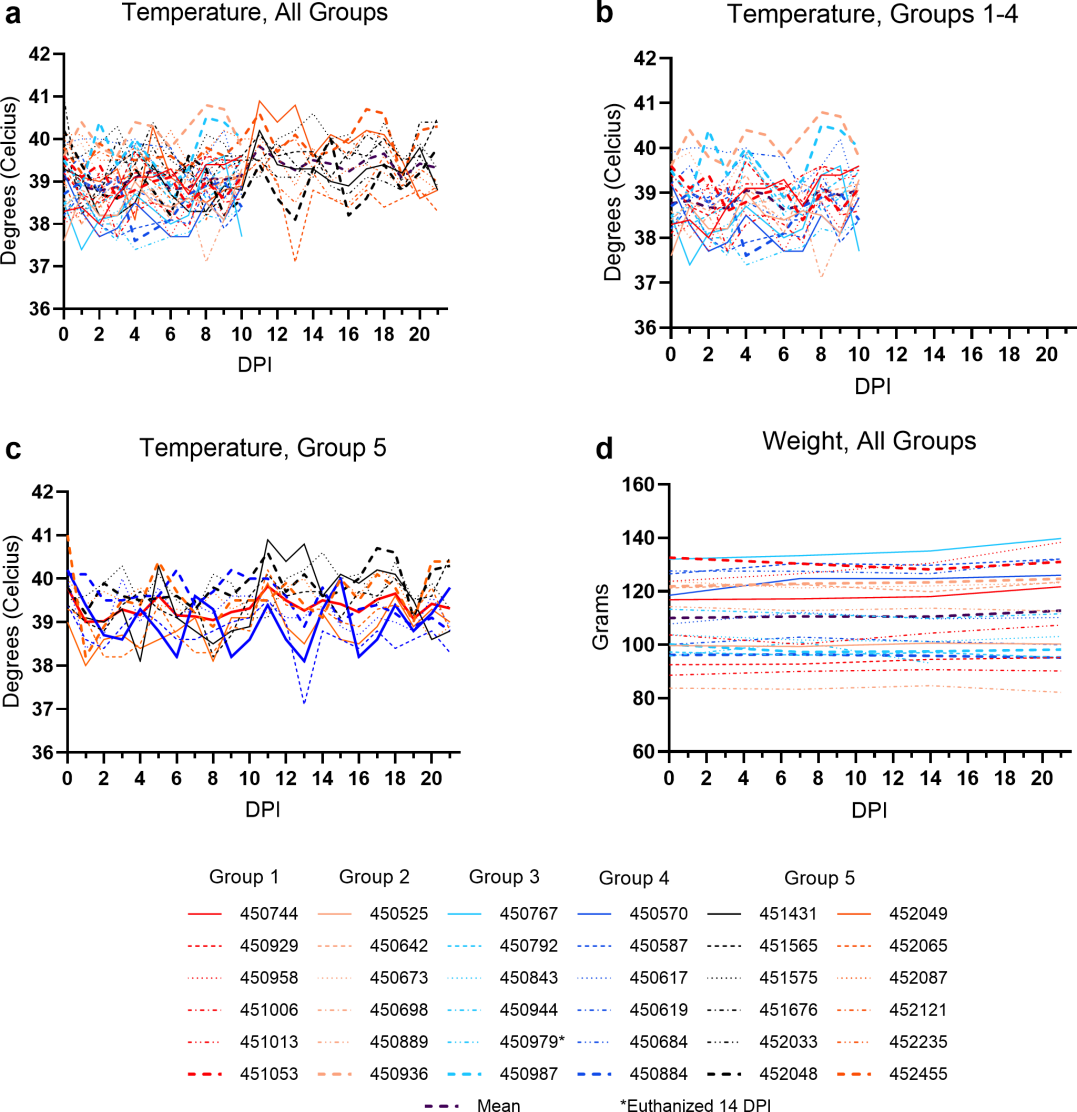
Clinical data. Temperatures (°C) acquired via rectal thermometer in (a) all bats, (b) groups 1–4, (c) Group 5, and (d) weights (grams) from experimentally infected ERBs. The purple dashed lines in a–d represent the overall mean value of each parameter. Bat 450979 was euthanized 14 DPI due to weight loss and declining health. DPI = days post-inoculation.

As in previous studies (31, 32, 35, 36), MARV- and RAVV-inoculated bats did not have any disease-related morbidity or mortality, and normal social and feeding behaviors were maintained. One female bat (450979) was euthanized on 14 DPI due to declining health (multifocal alopecia, dehydration) and weight loss. Gross and histopathologic evaluation of the animal revealed superficial dermal trauma, mild adrenal cortical hyperplasia, and atrophy of brown fat, and the gastrointestinal system was largely devoid of digesta, with no evidence of an infectious or inflammatory process or neoplastic disease. Significant hepatocellular glycogenosis was present; however, this is a common incidental finding in frugivorous bats (42, 71). Taken together, the post-mortem findings are suggestive of social “bullying” and deprivation of access to food and water by group conspecifics. Female bats cooperatively monopolizing and defending food resources from other females has previously been described (72). Apart from this bat, all other bats had normal body weights and rectal temperatures, consistent with previous studies (Fig. 2) (36, 40). As such, additionally, histopathology was, thus, deemed not necessary for any other bats.

## DISCUSSION

This study demonstrates that ERBs experimentally inoculated with homotypic MARV or heterotypic RAVV isolates mounted robust, fully protective secondary immune responses 236 days post-primary infection. This protective response occurred, regardless of whether the ERBs were initially monoinfected with MARV or coinfected with KASV + MARV. The immune response was characterized by the absence of orthomarburgvirus replication in the blood, no viral shedding from the oral or rectal mucosa, and a rapid, robust orthomarburgvirus-specific IgG antibody response by 7 DPI across all groups. These findings align with previous studies investigating both short- (48 DPI) (31) and long-term (17–24 MPI) (33) immune responses in MARV-monoinfected ERBs following homotypic inoculation.

Coinfection within a host involves complex interactions between pathogens, including competition for resources, modulation of immunological pathways, or the production of chemical compounds (73). Coinfecting viruses can lead to a variety of outcomes, such as viral interference, synergy, or noninterference (73–75). For instance, murine coinfection with ectromelia virus (ECTV) and lymphocytic choriomeningitis virus (LCMV) demonstrated that LCMV-induced type I interferons attenuated ECTV-induced disease while simultaneously weakening the immune response to LCMV, highlighting the complex, bi-directional effects of viral coinfections on immunity and disease (76). Similarly, cattle coinfected with two foot-and-mouth disease virus (FMDV) isolates showed variable outcomes, ranging from transient protection to severe disease, depending on the timing of inoculation (77). Viral recombination in these superinfected animals suggests that persistently infected hosts may contribute to the emergence of new FMDV isolates (77).

Mathematical models using susceptible (S), exposed (E), infectious (I), and recovered (R) compartments (SEIR models) have been employed to estimate orthomarburgvirus transmission patterns in ERB populations (78). Using this model, a closed population of 40,000 ERBs with a biannual birth pulse and 21-day latent period predicted an active MARV infection prevalence of less than 2.0%, closely matching the 2.5% observed in previous ecological studies (13) and is corroborated by serological data from MARV experimental studies in managed care ERBs (31, 32, 35, 36). While the SEIR framework is useful for modeling filovirus ecology, additional ecological and biological factors likely shape reservoir host and infection trajectories (e.g., metapopulation dynamics, the duration of protective immunity, natural immune stressors, and the role of coinfection in shaping susceptibility patterns and immune responses) (37, 79). Whether coinfection in ERBs alters the duration of immunity or hastens the return to susceptibility remains unknown but carries significant implications for viral persistence and transmission in free-ranging host populations.

In the previous experimental coinfection study from which our group 1–4 bats were derived, ERBs coinfected with KASV + MARV had significantly increased peak magnitude and duration of MARV viremia and oral shedding and had significantly higher cumulative MARV shedding loads (19). This implies a positive or synergistic interaction in which KASV infection facilitates or potentiates MARV infection. KASV infection did not appear to hinder the development of a robust MARV-specific immune response, as all MARV-inoculated ERBs had sterilizing immunity identical to previously monoinfected ERBs (31, 33). Interestingly, in the previous experimental coinfection study, only 10/12 of the ERBs coinfected with SOSV + MARV seroconverted to MARV by the end of the study (18 DPI), and the cohort had significantly lower anti-MARV nucleoprotein IgG responses compared to the MARV-monoinfected group (19). These findings raise the possibility that, unlike KASV + MARV coinfection, SOSV + MARV coinfection in ERBs might confer susceptibility to MARV reinfection, supported by the 2.5% annual prevalence of active MARV infection in adult ERBs (13). The possibility of increased susceptibility to MARV reinfection in adulthood is likely not limited to SOSV + MARV coinfection, as populations of ERBs are known to harbor multiple paramyxoviruses (80). Future experimental studies are needed to investigate long-term immune outcomes in ERBs coinfected with various viruses, including SOSV, as this study specifically examined KASV + MARV coinfection. Such studies should include investigating the impact of inoculation order and variation of inoculation intervals on MARV replication and shedding. Variation in inoculation time intervals has been shown to have wide-ranging effects on susceptibility in the context of viral (75, 81) infections and in subsequent immune responses (82). As such, further work is needed to elucidate these topics in the context of the ERB as a host of diverse and taxonomically varied microorganisms (19).

Compared to the results reported in this current study, previous investigations of long-term antibody dynamics in ERBs have yielded conflicting data. One study observed that virus-specific IgG levels in MARV-monoinfected ERBs declined rapidly after the initial infection peak, falling below the threshold of seropositivity by 3 months after infection, though a robust secondary immune response was observed upon homotypic inoculation 24 months later (35). In contrast, another study found that 67% of bats experimentally infected with MARV retained detectable antibodies around 4 MPI, while 84% of naturally exposed bats captured in the wild showed MARV antibodies at least 11 months later (83). While some MARV-naturally infected ERBs became viremic upon heterotypic inoculation, widespread tissue dissemination was not observed (83). Future research is needed to better understand the duration of protective immunity, especially in the context of previous coinfections that may affect differential immunity depending on the specific coinfecting agent (e.g., KASV + SOSV).

Overall, we have demonstrated that both prior MARV monoinfection and KASV + MARV coinfection in ERBs confer a consistent and robust secondary immune response that prevents reinfection upon viral inoculation with homotypic or heterotypic orthomarburgvirus isolates, MARV or genetically diverse RAVV, respectively. These findings broaden our understanding of the factors that influence this bat species' susceptibility to and maintenance of natural infections in free-ranging populations. Furthermore, these results offer new insights into immune dynamics in nature, where viral coinfections are common, and contribute to our understanding of how such interactions might impact the transmission of zoonotic pathogens from bats to susceptible hosts, including humans.
